# Differential regulation of transposable elements (TEs) during the murine submandibular gland development

**DOI:** 10.1186/s13100-021-00251-1

**Published:** 2021-10-22

**Authors:** Braulio Valdebenito-Maturana, Francisca Torres, Mónica Carrasco, Juan Carlos Tapia

**Affiliations:** 1 Núcleo Científico Multidisciplinario, School of Medicine, University of Talca, Campus Talca, Talca, Chile; 2 Stem Cells and Neuroscience Center, School of Medicine, University of Talca, Campus Talca, Talca, Chile

**Keywords:** Submandibular gland, Transposable elements, Gene regulation, Organogenesis, Development

## Abstract

The submandibular gland (SG) is a relatively simple organ formed by three cell types: acinar, myoepithelial, and an intricate network of duct-forming epithelial cells, that together fulfills several physiological functions from assisting food digestion to acting as an immune barrier against pathogens. Successful SG organogenesis is the product of highly controlled and orchestrated genetic and transcriptional programs. Mounting evidence links Transposable Elements (TEs), originally thought to be selfish genetic elements, to different aspects of gene regulation in mammalian development and disease. To our knowledge, the role of TEs during murine SG organogenesis has not been studied. Using novel bioinformatic tools and publicly available RNA-Seq datasets, our results indicate that a significant number of genic and intergenic TEs are differentially expressed during the SG development. Furthermore, changes in expression of specific TEs correlated with that of genes involved in cellular division and differentiation, critical aspects for SG maturation. Altogether, we propose that TEs modulate gene networks that operate during SG development.

**Supplementary Information:**

The online version contains supplementary material available at 10.1186/s13100-021-00251-1.

## Introduction

The salivary glands are responsible for producing buccal fluid for digestion, vocalization, oral pH maintenance and bacterial control [1, 32]. The submandibular gland (SG) is the largest of the salivary organs, it accounts for ~ 80% of the buccal fluid production. For the SG to achieve successful organogenesis, several steps must occur during its development [2, 20]. First, in mice, at E11 (embryonic day 11), a primordial thickening of the oral epithelium occurs (pre-bud). Second, at E12.5, the pre-bud begins to invaginate forming a primary bud, the structure that gives rise to a rudimentary ductal network. Third, at E16, the canalicular ducts begin to form and branch profusely to generate a denser ductal system. Also, at this stage, the acini main organization begins to appear. Fourth, at E18, numerous acini commence to associate with a more intricate embryonic ductal network. Fifth, at birth (P1, postnatal day 1), the SG becomes fully functional although growth continues for approximately 4 weeks (P28), reaching complete acinar maturation at P70.

An exquisite coordination between genetic and environmental factors are responsible for converting the SG pre-bud stage into its mature form [20], and thus several gene expression studies have been performed to analyze the murine SG development [10, 21, 22]. About 2000 genes have been found to be differentially expressed by the murine salivary glands, with about 700 of them exclusive to the submandibular gland, when compared to the parotid and sublingual salivary glands [10]. Unfortunately, most of these studies were performed using microarrays, a technique that has limited range of gene detection (i.e., mostly biased to known sequences), among other complications [35]. Gluck et al. [11] pioneered the use of RNA-Seq for studying the temporal course of the murine SG transcriptome. This technical advance offered the possibility of studying changes in transcriptional regulation with nucleotide-resolution (i.e., significantly higher resolution than microarrays), and importantly, allowed the analysis of elements that participate in gene regulation at the transcriptional level [35]. Thus, Gluck et al. were not only able to confirm the activity of previous genes known to be of importance for the SG; in addition, they found a series of novel elements that regulate transcription. Moreover, the authors generated a gene and signaling pathway signature for each developmental stage, revealing that indeed SG organogenesis required the coordination of highly controlled genetic and transcriptional programs [11].

In spite of the significance of the work for understanding SG organogenesis, Gluck et al., did not analyze the expression of Transposable Elements (TEs). TEs are genetic elements with the ability to move within the genome by a copy-and-paste mechanism (class I TEs, retrotransposons) or via a cut-and-paste mechanism (class II TEs, DNA transposons) [15]. Class I TEs are mainly subdivided in the LINE, SINE and LTR groups, and each of these groups are thought to influence gene expression in different ways [8]. Regardless of the categorization, as a product of their activity, TEs are highly repetitive, and represent about ~ 50% of the mouse genome. Because of the potentially deleterious consequences of this activity, most TEs have suffered mutations that render them inactive, with only a few copies being able to transpose. Nonetheless, some TEs are still transcriptionally active, and they can influence gene activity in neighboring genes or in genes located far away in the genome [6]. Thus, it is now well accepted that TEs either by their transposition or by their transcriptional activity play roles in gene regulation [15]. Thus, for example, transcriptional activity of some TEs can impede transcription of genes, by interrupting Polymerase II activity, among other mechanisms [8]. Overall, TE activity has been implicated in several cellular regulatory processes in both health and disease [6].

In general, TEs are not routinely studied in RNA-Seq experiments. This is because the tools available to estimate their expression levels lose information regarding the TE locus, preventing understanding of possible events of gene regulation by TEs. Recently, the TEcandidates [34] and SQuIRE [37] tools were developed to allow locus-specific estimation of TEs expression. Therefore, these bioinformatic tools make it possible to investigate what TEs are expressed in RNA-Seq datasets and allows subsequent analyses to determine whether their expression levels correlate with that of particular genes.

To our knowledge the expression of TEs and their putative role during SG organogenesis has not been examined. Thus, the objectives of our work were two: first, to determine if TEs were expressed during SG development; and if so, second, to establish the putative regulatory influences of TEs on the expression of specific genes that may be involved directly or indirectly in SG organogenesis. To achieve these goals, we performed TEs expression analyses with the previously mentioned tools, studying the diversity and regulatory role of thousands of TEs. Then, using statistical analysis we evaluated the correlation between selected TEs and gene expression. Putting the results all together, we argue that TEs are indeed implicated in SG development.

## Results

### TEs expression during submandibular gland (SG) development

**Fig. 1:**
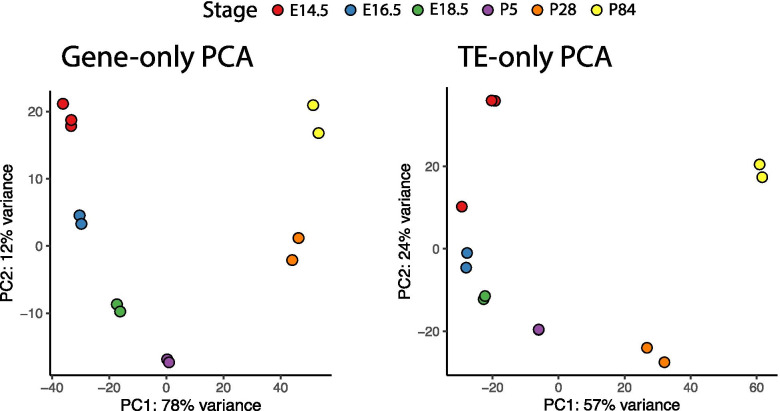
PCA plots using the gene expression levels (**left**) and TE expression levels (**right**). Points are colored according to their stage: Red, E14.5; Blue, E16.5; Green, E18.5; Purple, P5; Orange, P28; Yellow, P84

Our first goal was to assess whether TEs were expressed during the murine SG organogenesis. To do this, we took advantage of the most comprehensive RNA-Seq data available during SG development [11]. Thus, our bioinformatic analyses were based on 15 RNA-Seq datasets obtained at different stages of SG development, which were listed as follows: 3 datasets obtained on embryonic day E14.5, 2 datasets from E16.5 and 2 of E18.5. We also used datasets obtained at different postnatal ages, specifically 2 from P5, 2 from P28, 2 from P84 and 2 of P144. To determine the locus-specific transcriptional activity of TEs as a function of SG development, we used the bioinformatic tools SQuIRE and TEcandidates (see Methods). First, we performed PCAs using gene expression and TE expression (Fig. 1). With this analysis, we found that the PCA performed with TE expression follows a similar trend to the one based on gene expression, indicating that TEs are expressed at different timepoints of SG development (Fig. 1). To gain an overall understanding of the changes in TE expression, we then performed differential expression analysis for each developmental stage and compared their expression levels with respect to E14.5.

**Fig. 2:**
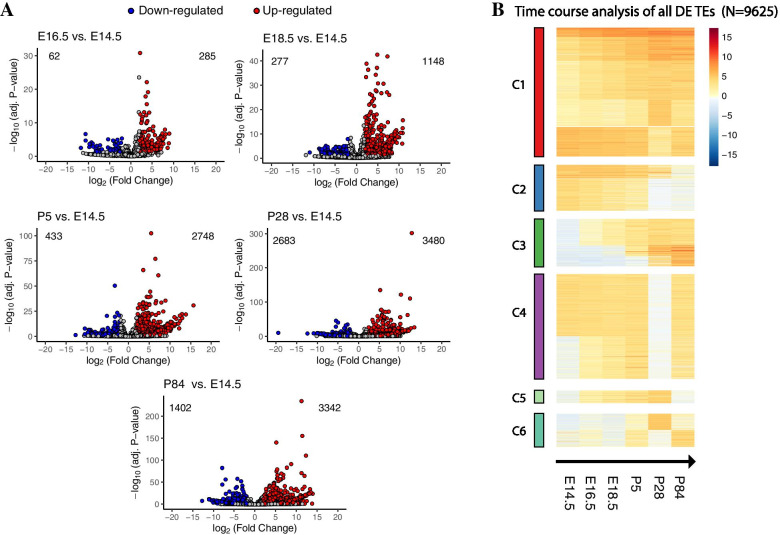
Total TEs expression at different SG developmental stages. **A**. Volcano plots depicting comparisons for all SG developmental stages, from E16.5 onwards, all with respect to E14.5. For each volcano plot, up- and down- regulated TEs are shown in red and blue circles respectively; gray circles indicate all TEs without statistically significant changes in expression. Upper-left and upper-right numbers for each volcano plot indicate the total number of down-regulated (blue) and up-regulated TEs (red), respectively. **B**. Heatmap depicting differentially expressed (DE) TEs (*N* = 9625) during all SG developmental stages. TEs were clustered according to their patterns of expression using the k-means clustering. Different clusters of expression are indicated (C1 to C6), color coded at the left of the heatmap. The range of expression values is shown to the right (red, positive values; blue, negative values). All developmental stages, from embryonic (E) to postnatal (P) days, are indicated in chronological order, and the arrow indicates direction of development

Stage-specific comparisons shown as volcano plots (Fig. 2A) revealed that relative to E14.5, there was a significant increase in the number of TEs differentially expressed throughout the SG development (Table 1). While a prominent number of TEs showed a decrease in the expression levels as the SG advanced in maturation (Fig. 2A, blue circles), another set of TEs showed increased levels of expression (Fig. 2A, red circles). Despite of these changes, many TEs were not altered at all, maintaining a relatively constant expression throughout the SG organogenesis (Fig. 2A, gray circles).

**Table 1:** Number of up-regulated and down-regulated TEs at each comparison done with respect to E14.5

Comparison	Up-regulated TEs	Down-regulated TEs
**E16.5 vs E14.5**	285	62
**E18.5 vs E14.5**	1148	277
**P5 vs E14.5**	2748	433
**P28 vs E14.5**	3480	2683
**P84 vs E14.5**	3342	1402

To better understand the expression profile of the differentially expressed (DE) TEs, we organized them in a clustered heatmap (Fig. 2B). Overall, a total of 9625 (out of 47,333 expressed TEs) were differentially regulated. The DE TEs can be grouped in 6 clusters according to their expression patterns: C1 – High expression, with small changes towards the end of SG development, C2 – High expression at early stages, with an important reduction in their levels at later stages (P5 onwards), C3 – low expression at early stages, but with higher levels at later stages, C4 – low expression at P28, C5 – high expression from E16.5 up to P28 and C6 – oscillatory patterns of expression (Fig. 2B).

In sum, our results showed a clear change in the expression of TEs during the entire murine SG development, with several TEs increasing their activity.

### Genic and intergenic TEs expression during submandibular gland (SG) development

**Fig. 3:**
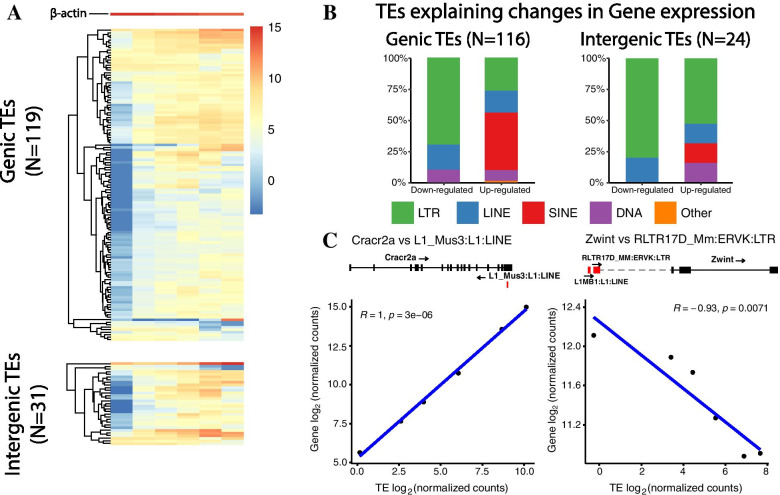
Expression and statistical analysis of TEs predicted both by SQuIRE and TEcandidates. **A**. Heatmap of log_2_(normalized counts) of selected TEs. At the top of the heatmap, the housekeeping gene β-actin is shown as a reference. The heatmap was divided into genic TEs (overlapping genes) and intergenic TEs (not overlapping genes). **B**. Class distribution of genic TEs and intergenic TEs that explain changes in gene expression with statistical significance (*p* ≤ 0.05). **C**. Two plot examples of Gene vs TE log_2_(normalized counts), sorted by stage (i.e., from left to right, each point shows the gene and the TE normalized count at E14.5, E16.5, and so on). Each plot shows the R coefficient of correlation, and its corresponding *p*-value, and in blue, the regression line. The left plot shows an example of a positive correlation, and the right plot an example of a negative correlation

To investigate further our results, we analyzed the TEs that depicted changes in expression, predicted both by SQuIRE and TEcandidates. This selection resulted in a total of 150 TEs (Fig. 3), which were labeled as either genic (within the gene body, Fig. 3A, top) or intergenic (outside a gene, Fig. 3A, bottom). Such locus-specific classification revealed that 119 were genic and 31 TEs were intergenic. Of these, we found that 17 genic TEs and 6 intergenic TEs showed marked increase in their levels of expression at all developmental stages after E14.5 (Fig. 3A). This was clearly observed when TEs were compared with the expression of β-actin, a well-known housekeeping gene that remained constant during all SG organogenesis.

We then analyzed the classes of TEs present during SG development based on the direction of gene expression change (i.e., up- or down-regulation) (Fig. 3B). We found that amongst the genic TEs, the most prevalent up-regulated TEs were of the SINE (46%) and LTR classes (26%), whereas amongst the down-regulated, the most prevalent were of the LTR (70%) and LINE classes (20%), with few of them belonging to the DNA class (10%) (Table S2). For the intergenic TEs, the majority corresponded to the LTR type (53% amongst the up-regulated, and 80% amongst the down-regulated), with few TEs belonging to the SINE, LINE and DNA groups (Table S3). Compared to the global TE genome proportions (42% SINE, 27% LINE, 26% LTR and 4% DNA), for genic TEs there were an statistically significant decrease in the proportion of up-regulated LINE TEs (*p* < 0.05, Table S2), and a statistically significant increase in the proportion of down-regulated LTR TEs (Table S2). For intergenic TEs, we observed a marked and significant increase in the proportion of LTR TEs regardless of the direction of gene expression change (> 2 times the original composition (Table S3) and a significant decrease of up-regulated SINE TEs, with all the other types showing not statistically significant changes in their proportions (Table S3). Overall, these results indicated that most differentially expressed TEs were retrotransposons, a class well known to be involved in gene regulation [8].

To assess the potential modulatory effect of TEs on gene expression, we first associated TEs with genes based on their genomic location (Additional File 2, Fig. S2). Genic TEs were associated to the gene with which they overlapped (“host gene”), whereas the intergenic TEs were associated to their closest downstream gene. Afterwards, we used TEffectR [13] to assess the statistical association between TEs and their respective genes (Methods). This resulted in 116 genic TEs and 24 intergenic TEs (mean distance to their closest downstream gene: 51,941 bp, Additional File 3) that were statistically associated with genes. Additionally, we calculated the expected proportion of TEs in the context of changes in gene expression (Methods), which resulted in 56%. Here, the proportion was 93.3% (140 out of 150), increment that was statistically significant (*p* < 2.2e-16), suggesting that TE expression and the gene expression are highly intertwined. To assess whether the potential modulatory effect of TEs on their associated genes could be positive or negative, we performed pairwise TE - gene correlations. We found that 90 genic and 13 intergenic TEs strongly correlated with the levels of expression of their respective associated genes (Additional File 4). Using a stringent criteria of statistical significance (Methods), we found that of these, 81 genic TEs positively correlated with their respective host genes, while 9 of them showed negative correlation with the host genes. Examples of statistically significant correlations are shown for the genes Cracr2a (positive correlation) and Zwint (negative correlation) in Fig. 3C. We also found that 7 intergenic TEs positively correlated with their respective closest downstream genes, while 6 intergenic TEs showed negative correlation. These results were consistent with a potential modulatory role of genic and intergenic TEs on their respective associated genes. Since we were unable to distinguish whether the positive correlation of genic TEs with their host genes was due to transcription driven by the TE or by its host gene, we labeled these events as co-transcription.

### Gene targets potentially regulated by TEs during SG development

**Fig. 4:**
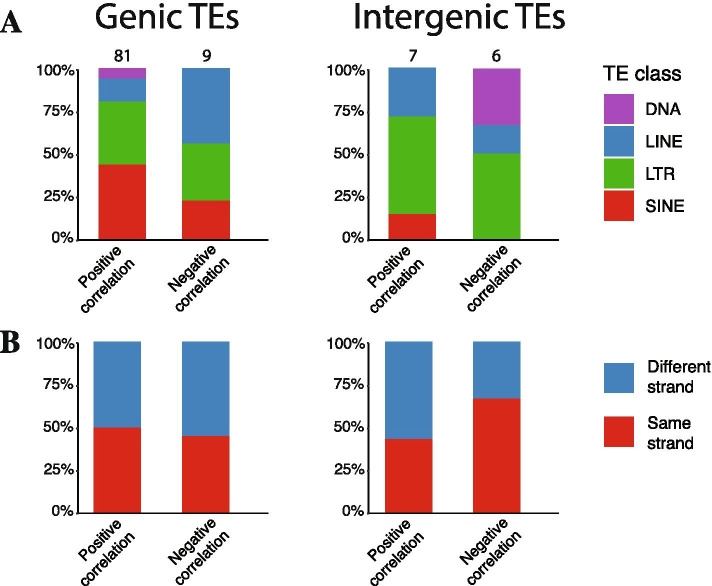
Feature analysis of TEs with significant correlation with genes. A. TE class distribution (DNA: Purple, LINE: Blue, LTR: Green, SINE: Red). B. TE strand distribution (Blue: TEs in different strand that its associated gene, Red: TEs in the same strand than its associated gene)

To identify genes that could be regulated by genic and intergenic TEs and learn about their putative physiological contribution during the SG development, we analyzed gene expression that correlated with that of the TEs (R ≥ 0.8). We found 81 genic TEs that correlated positively with their associated gene, and 9 genic TEs that correlated negatively with their associated gene (Fig. 4A). In the case of intergenic TEs, we found 7 TEs correlating positively with their respective associated genes, and 6 TEs correlating negatively with their respective associated genes (Fig. 4B). In addition, we found no strand bias when analyzing each of the correlation groups, except for the intergenic TEs that showed a bias towards those located in the same strand of their closest downstream genes when they were positively correlated, an association that could be related to transcriptional repression mediated by transcription of intergenic elements [24]. Within all pairs selected, some genes that strongly correlated with the TEs expression did not have a defined biological process associated to them. This was the case for 18 out of 57 (31.2%) genes associated with genic TEs, and 5 out of 12 (41.2%) of the closest downstream genes of intergenic TEs (Additional File 4), turning difficult to predict the impact of their potential regulation by TEs during SG organogenesis. Amongst the genes whose expression correlated negatively with that of their associated TEs, we found genes participating in cell differentiation and transcription (for example: Ehf, Elf5, Appl2 [18], and Cldn10) (Additional File 4), further suggesting that TEs could play a key role during this biological process.

## Discussion

TEs, originally described as junk DNA, are now considered important regulatory players, modulating gene expression during different cellular processes [6]. Thus, the purpose of this work was to determine whether TEs were present during SG development, and if so, examine their putative role in regulating gene expression. By analyzing RNA-Seq datasets for the SG at different stages, we found that a large repertoire of TEs (> 9000) were differentially expressed throughout the SG development. Interestingly, some of the differentially expressed TEs showed significant changes in their patterns of expression, suggesting additional interactions during SG development. We then characterized a subset of TEs predicted by the combination of both SQuIRE and TEcandidates (*N* = 150).

We then characterized a subset of TEs predicted by the combination of both SQuIRE and TEcandidates (*N*=150). According to their genomic locus, genic and intergenic TEs were identified, which mainly belonged to the LTR, LINE and SINE subclasses. By linking TEs with genes (using a strong correlation coefficient R ≥ 0.8), we found that 81 of the 90 genic TEs have positive correlation values, which could be indicative of co-transcription events (i.e., TEs transcribed as a consequence of its host gene being transcribed) [15]. On the other hand, the remaining 9 genic TEs have negative correlation values with their host gene, which might be related to transcriptional interference (i.e., TE transcription interrupting the host gene normal transcription) [9]. In terms of genes, we identified 68 that could potentially be subjected to regulation by TE expression. Although several of the genes associated with TEs did not have a known biological process, a few can be associated with cell differentiation. Among these genes, we highlight Elf5. This gene has been found in one of the two epithelial lineages of the SG, as well as in other organs [5, 16]. Moreover, Elf5 has been found to be critical since Elf5-null embryos die early during embryogenesis, exhibiting severe ectodermal defects [38].

It is worth noting that due to the stringent correlation cutoff (R ≥ 0.8) used, we discarded potential TE-mediated gene regulation events that might be subtle, but biologically relevant. For example, Tspan15 and Cntn1 were genes just below the threshold (R ~ 0.7). These genes have been associated with Notch signaling, which is implicated in a variety of biological processes such as cell fate, differentiation, proliferation, and organogenesis. Thus, we do not discard that TEs may play an even larger role during SG organogenesis.

In addition, we found a small overlap between the DE TEs and regulatory elements such as enhancers (Additional File 2, Fig. S5 and Table S1), further suggesting that these TEs might be involved in gene regulation. Moreover, as TEs can also mediate epigenetic repression of neighboring genes [6], future works need to explore their role in SG development at the epigenetic level. Although hinted, our work suggests that TEs may regulate genes at the single-cell level. Thus, future analyses with cellular resolution might help us understand the role of TEs in the specification of particular cell populations.

A caveat for detecting coding and non-coding transcripts (i.e., such as TEs) is that of the type of library used for sequencing, which entails either ribosomal RNA (rRNA) depletion (removal of highly abundant rRNAs) or poly-A selection method containing all polyadenylated mRNAs (used by Gluck et al. [11], and thus in our analysis). Most TEs are transcribed by RNA Polymerase (Pol) II [6, 29], and its well accepted that Pol II transcripts are polyadenylated upon recognition of the polyadenylation signal (PAS) [25, 30]. Indeed, LINE TEs are polyadenylated, and intact LTR TEs carry a PAS at their 3′ UTR [6, 29]. The SINE TEs are an exception, because these TEs are commonly transcribed by Pol III [19, 23]. However, there is evidence that some SINEs can be polyadenylated in a similar way as Pol II transcripts [3, 28, 33]. Thus, we argue that by using the Gluck et al. dataset, we might only under-represent SINE TEs and few other non-polyadenylated TEs. To get a real estimation in terms of the differences in reads that map to TEs using these two methods of library preparation for RNA-Seq (i.e., poly-A RNA selection or ribosomal RNA depletion), we analyzed several publicly available datasets in which both protocols were used for mammalian cell lines grown in vitro and for tissue samples (Additional File 6). Overall, we observed an increase in the percentage of reads mapping to TEs of 3.2–13.2% when using rRNA depletion methods vs the polyA selection method (Additional File 6). Thus, we argue that while the use of poly-A RNA-Seq libraries might not be ideal, it is capable of retaining more than 70% of all TEs (Fig. S6). Altogether, our results support the view that TEs might modulate gene networks underlying SG organogenesis. Follow-up experimental approaches will test this hypothesis.

## Methods

### RNA-Seq datasets and availability

The RNA-Seq dataset utilized in this study was previously published by Gluck et al. [11], and is publicly available at the Gene Expression Omnibus database, accession number GSE81097. The RNA-Seq datasets for all experimental conditions were performed in an Illumina HiSeq 2500 platform, resulting in single-end reads 50 bp in length. A summary of the number of reads per library is shown in Table 2.

**Table 2:** Number of reads per RNA-Seq library published by Gluck et al. [11]

Run	Age	Number of reads
**SRR3475588**	E14.5	24,187,535
**SRR3475589**	E14.5	31,175,495
**SRR3475590**	E14.5	33,384,282
**SRR3475591**	E16.5	46,682,402
**SRR3475592**	E16.5	48,796,458
**SRR3475594**	E18.5	41,930,729
**SRR3475593**	E18.5	22,866,594
**SRR3475599**	P5	67,221,144
**SRR3475600**	P5	57,435,611
**SRR3475597**	P28	33,371,615
**SRR3475598**	P28	20,499,918
**SRR3475595**	P84	50,918,269
**SRR3475596**	P84	54,710,880

“Run” corresponds to the SRA accession run identifier, “Age” corresponds to the timepoint (stage) at which the samples were sequenced, and “Number of reads” is equivalent to the total amount of reads in the sequenced library

### Bioinformatics analyses

In the work of Gluck et al. [11], the *Mus musculus* genome version mm9 was used, with reads aligned using TopHat2. Here, we used the mm10 genome with the corresponding gene and TE annotation files, and the STAR aligner for read mapping (see below). TE analysis was carried out using SQuIRE [37] and TEcandidates [34]. Both of these tools take as input the raw reads FASTQ files. First, we used SQuIRE to perform read alignment. SQuIRE calls STAR [7] with options suited for TE analysis, quantification of reads per gene, and estimation of reads per TEs in a locus-specific manner. Afterwards, we used TEcandidates to further confirm the expressed TE locus. TEcandidates was used with options Coverage = 0.1 and TE length = 900, explained next. Briefly, TEcandidates performs de novo transcriptome assembly to obtain in silico long reads, which can avoid multimapping ambiguity. These in silico reads are mapped to the reference genome, and the coverage of TEs by assembled reads is calculated. TEs having a coverage ≥0.1 (i.e., being covered by a de novo transcript in at least 10%), and length ≥ 900 are reported. The main drawback of TEcandidates is that it does not estimate the expression of TEs. Thus, for the next analysis, the expression of TEs estimated by SQuIRE was used. As final output, SQuIRE generates a raw count matrix of gene and TEs across all conditions. This count matrix was used for the subsequent analysis. Similarly to Gluck et al., read count normalization and differential expression analysis were performed using DESeq2 [17]. Differential expression analysis for genes and TEs were performed at ages E16.5, E18.5, P5, P28 and P84, using E14.5 as baseline. To select differentially expressed TEs (DE TEs) an adjusted *P*-value ≤0.05 and|log_2_(Fold Change)| ≥ 2 was used. DE TEs found at this step, were then analyzed in absolute terms across all time points using their respective log_2_(normalized counts). We performed k-means clustering with this data using the “kmeans” R function, to group the DE TEs according to their patterns of expression. The result of this analysis was then plotted as a heatmap using the pheatmap package [14] in the R statistical software [27].

For the second part of our work, we selected TEs that were predicted both by SQuIRE and TEcandidates. The subsets of TEs were selected for gene association analyses based on their locus. Briefly, the genomic overlap between these TEs and genes was assessed using bedtools intersect from BEDTools v2.29.2 [26]. TEs were classified into either genic TEs, if they had an overlap with genes, or intergenic TEs, if they didn’t have an overlap with genes. For intergenic TEs, an additional analysis was performed using bedtools closest, using as -a file the intergenic TEs, and -b file the genes, with options “-D a” to label genes as upstream or downstream relative to TEs, and “-iu” to ignore genes upstream of TEs. This allowed us to find only the subset of genes that were downstream of a TE. This new subset of TEs was plotted again as a heatmap, as described above, with the addition of β-actin for reference. Gene-TE pairs were then obtained according to the mentioned classification: for genic TEs, the gene with which each TE overlapped was assigned as its pair, whereas for intergenic TEs, the closest downstream gene was assigned as its pair, with no distance threshold.

Statistical analysis of gene – TE pairs was done in two rounds. First, we used TEffectR [13] to assess whether the gene expression could be explained by TE expression. Briefly, TEffectR takes as input the gene and TE count matrix, and list of gene-TE pairs. Then, the expression of a gene is modeled with a linear model, in which the corresponding TE expression (according to the gene-TE pair list) is used as a predictor variable. Through this modeling, TEffectR returns model *p*-values for each gene-TE pair, which can then be used to filter the most significant results. To calculate the expected number of TEs in context of changed gene expression, TEffectR was first used with all the DE TEs – gene pairs (10,011 in total), and a cutoff of model *p*-value ≤0.05. Then, TEffectR was used on the TE-gene pairs of TEs predicted by both TEcandidates and SQuIRE. The statistical significance of the difference between these proportions was calculated with the “prop.test” function of the R statistical software [27]. Afterwards, only gene-TE pairs with model *p*-value ≤0.05 were kept for the following round. The correlation of log_2_(normalized counts) of TEs and log_2_(normalized counts) of its respective gene across each time point, was then obtained using the “cor” function of the R statistical software [27]. Correlations with *p* ≤ 0.05 were only considered. Afterwards, we only kept correlations that were moderately strong (absolute value of correlation ≥0.8).

Unless otherwise noted, all plots were produced using R, with the ggplot2 package [36].

Predictions of gene biological processes were done with Gene Ontology [4], and complemented with annotations from UniProt [31] and Reactome Pathway database [12].

## Supplementary Information

**Additional file 1.** Locus-specific details of differentially expressed TEs across all time points.

**Additional file 2: Figure S1.** MA plots of differentially expressed (DE TEs). **Figure S2.** Schematic representation of how genes were attributed to a TE. **Figure S3.** TE Class distribution. **Figure S4.** Relative distribution of TEs with respect to their associated genes. **Figure S5.** Overlap of TEs with Promoters from the Eukaryotic Promoter Database (EPD) and RefSeq Functional Elements (RefSeqFuncElems). **Table S1.** Detailed information of the overlap of Differentially Expressed TEs (“TE”) with RefSeq Functional Elements (“RefSeq Functional Element”). **Table S2.** Proportion test results of the selected genic TEs in section 2 of our work versus the genomic TE distribution. **Table S3.** Proportion test results of the selected intergenic TEs in section 2 of our work versus the genomic TE distribution. **Table S4.** Enhancers within 52 kb of genes associated with intergenic TEs.

**Additional file 3.** Supplementary tables of genic and intergenic TEs and their respective associated genes.

**Additional file 4.** Supplementary tables of TE-Gene correlations, with each Gene biological process reported.

**Additional file 5.** Supplementary table of Elf5 binding sites search using Find Individual Motif Occurrences v5.3.3 in selected TEs.

**Additional file 6: Table S5.** Alignment statistics of the RNA-Sequencing samples published by Zhang et al. (2014). **Table S6.** Alignment statistics of the RNA-Sequencing samples published by Cui et al. (2010). **Table S7.** Alignment statistics of the RNA-Sequencing samples published by Zhang et al. (2018).. **Figure S6.** Venn diagram of the TEs identified by SQuIRE in the datasets published by Cui et al. (2010).

## Data Availability

The datasets analyzed during the current study were previously published, and they are publicly available in the GEO database, under the accession number GSE81097. All data generated or analyzed during this study are included in this published article [and its supplementary information files].

## Abbreviations

TE: Transposable Elements
SG: Submandibular Gland
