# Supplementary material for: Differential regulation of transposable elements (TEs) during the murine submandibular gland development
Source: Mob DNA. 2021 Oct 22;12:23. doi: 10.1186/s13100-021-00251-1 (PMC8540199; doi:10.1186/s13100-021-00251-1)
Supplement: Supplementary file 2 — Additional file 2: Figure S1. MA plots of differentially expressed (DE TEs). Figure S2. Schematic representation of how genes were attributed to a TE. Figure S3. TE Class distribution. Figure S4. Relative distribution of TEs with respect to their associated genes. Figure S5. Overlap of TEs with Promoters from the Eukaryotic Promoter Database (EPD) and RefSeq Functional Elements (RefSeqFuncElems). Table S1. Detailed information of the overlap of Differentially Expressed TEs (“TE”) with RefSeq Functional Elements (“RefSeq Functional Element”). Table S2. Proportion test results of the selected genic TEs in section 2 of our work versus the genomic TE distribution. Table S3. Proportion test results of the selected intergenic TEs in section 2 of our work versus the genomic TE distribution. Table S4. Enhancers within 52 kb of genes associated with intergenic TEs. [file 13100_2021_251_MOESM2_ESM.docx]

**SUPPLEMENTAL INFORMATION**

**Differential regulation of TEs during murine Submandibular Gland Development**

Braulio Valdebenito-Maturana, Francisca Torres, Mónica Carrasco^*^, Juan Carlos Tapia^*^

**Information in this document:**

Figure S1

Figure S2

Figure S3

Figure S4

Figure S5

Table S1

Table S2

Table S3

Table S4


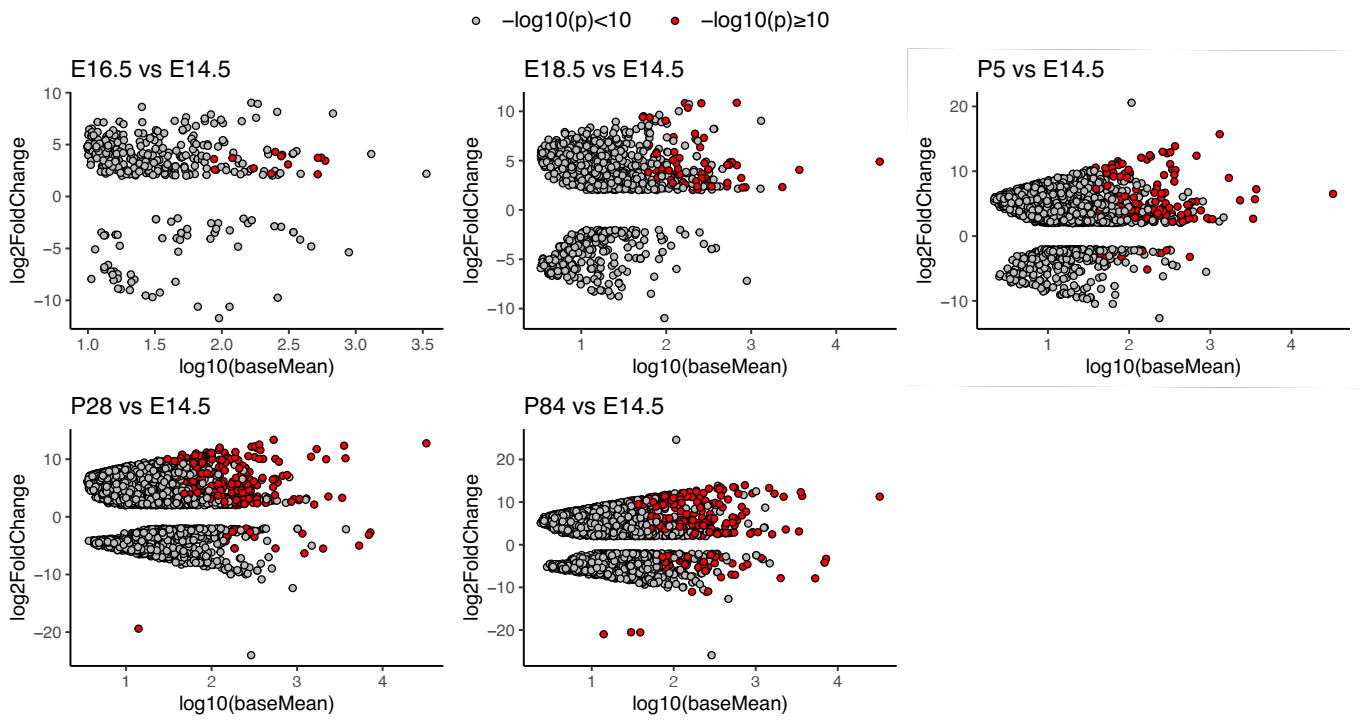


**Figure S1. MA plots of differentially expressed (DE TEs).** The mean abundance (base mean) is shown in log10 scale across the x-axis, and the fold change in log2 scale is shown across the y-axis. Points are colored according to their adjusted p-value (p) in -log10 scale.


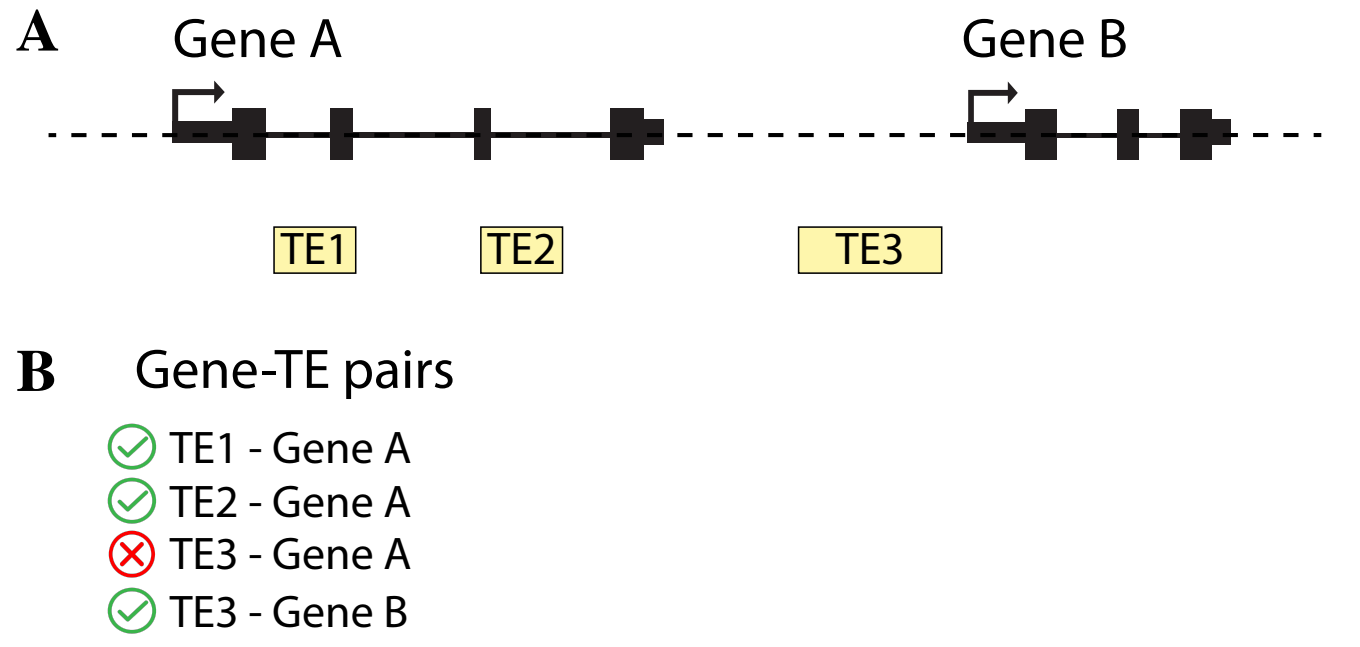


**Figure S2. Schematic representation of how genes were attributed to a TE.** A. Two genes are depicted along a hypothetical genome. The black rectangles in each gene corresponds to their respective gene body, whereas the solid black line connecting them represents their introns. 3 TEs are shown, with TE1 and TE2 being in the introns of Gene A, and TE3 being in an intergenic region, upstream of Gene B. B. According to their position relative to each gene, TEs are associated with a gene in the following way: if a TE is inside a gene, it is associated to that gene; if a TE is in an intergenic region it is associated to the closest downstream gene. In this example, TE1 and TE2 are associated to Gene A, whereas TE3 cannot be associated to Gene A, as its closest downstream gene is Gene B.


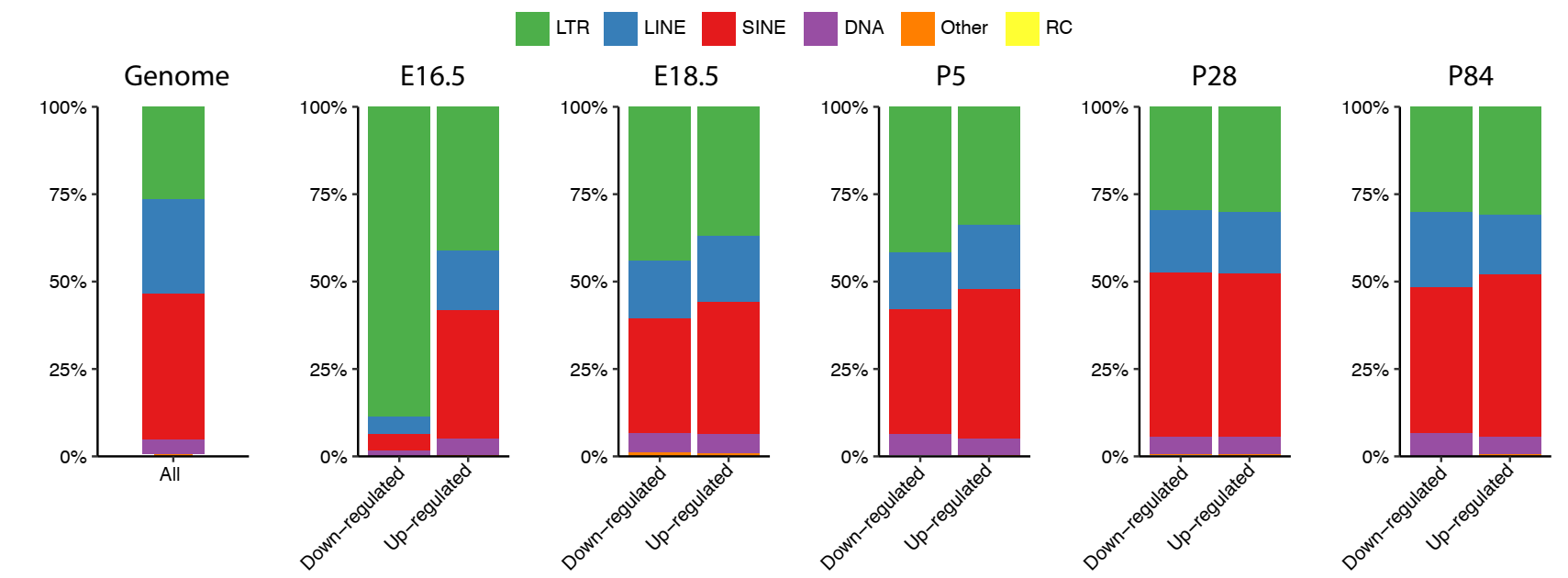


**Figure S3. TE Class distribution.** The background / genome distribution is shown in the first bar, and the distribution at each time point is shown in the subsequent bars. For these bars, the left one corresponds to the down-regulated TE class distribution, whereas the right one to the up-regulated TE class distribution.


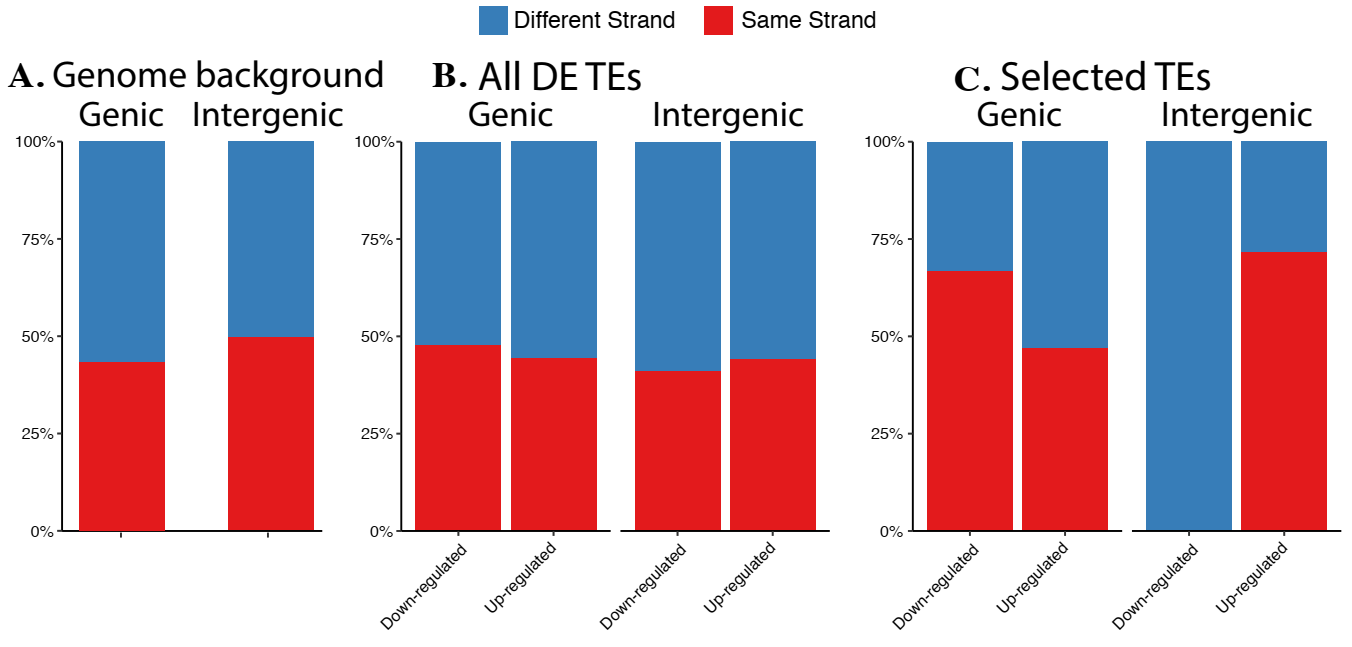


**Figure S4. Relative distribution of TEs with respect to their associated genes.** A. Genome background, B. All Differentially Expressed TEs (N=9625), C. Selected TEs (N=150). The proportion of TEs being on different strand than their associated genes is shown in blue, whereas the proportion of TEs being on the same strand as their associated genes is shown in red.


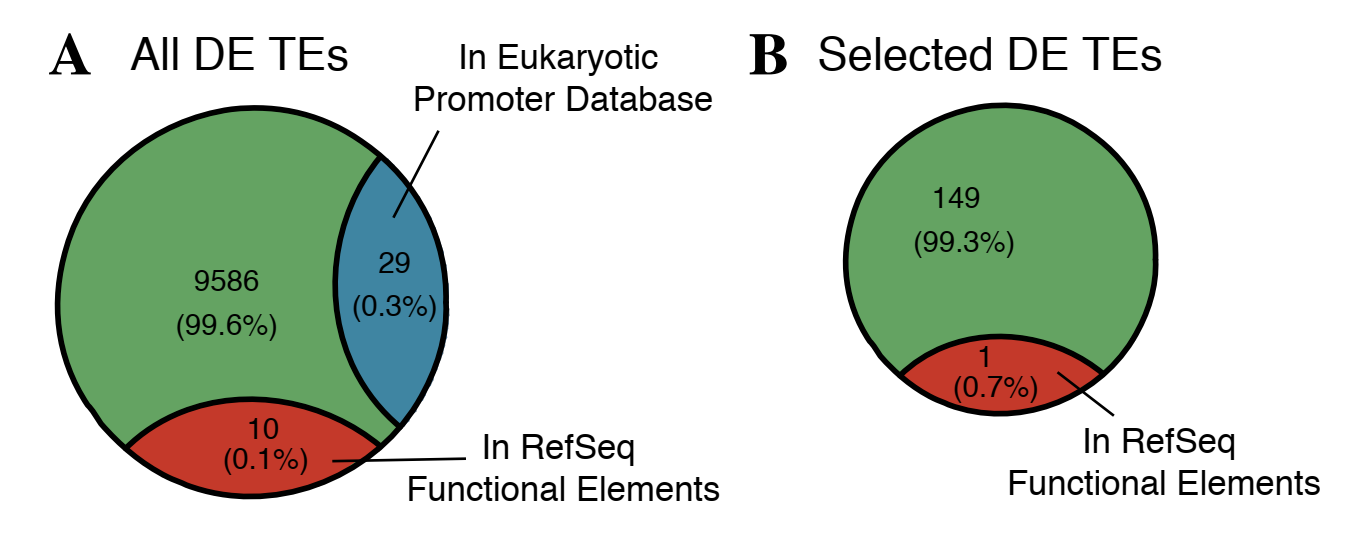


**Figure S5. Overlap of TEs with Promoters from the Eukaryotic Promoter Database (EPD) and RefSeq Functional Elements (RefSeqFuncElems).** A. Venn diagram of all Differentially Expressed (DE) TEs overlapping EPD Promoters or RefSeqFuncElems. B. Venn diagram of selected DE TEs overlapping EPD Promoters or RefSeqFuncElems. Partial Venn diagrams are shown because when overlapping genomic intervals using BEDTools the results are given relative to one file, in our case, the TE files.

| **TE** | **RefSeq Functional Element** | **Description** |
| --- | --- | --- |
| chr1\|133350196\|133350598\|RMER17B2:ERVK:LTR\|206\|- | silencer | 340 nt XbaI fragment |
| chr1\|133350196\|133350598\|RMER17B2:ERVK:LTR\|206\|- | promoter | AluI/EcoT14I fragment in mRN224CAT |
| chr1\|133350196\|133350598\|RMER17B2:ERVK:LTR\|206\|- | transcriptional_cis_regulatory_region | RU-1 element (-224 to -138) |
| chr1\|133350196\|133350598\|RMER17B2:ERVK:LTR\|206\|- | protein_bind: nuclear factor I C | RNFIU |
| chr1\|133350196\|133350598\|RMER17B2:ERVK:LTR\|206\|- | transcriptional_cis_regulatory_region | -197 to -70 |
| chr1\|133350196\|133350598\|RMER17B2:ERVK:LTR\|206\|- | protein_bind: Sp1/Sp3 transcription factors | RCACC |
| chr1\|133350196\|133350598\|RMER17B2:ERVK:LTR\|206\|- | protein_bind: nuclear factor I C | RNFID |
| chr1\|133350196\|133350598\|RMER17B2:ERVK:LTR\|206\|- | promoter | 4.1 kb fragment (-4100 to +6) |
| chr11\|11956763\|11956995\|Tigger7:TcMar-Tigger:DNA\|284\|- | enhancer | VISTA enhancer mm154 |
| chr11\|33150663\|33150803\|B1_Mm:Alu:SINE\|211\|+ | promoter | 3.3 kb MscI fragment |
| chr17\|34825294\|34825783\|LTRIS4:ERV1:LTR\|61\|+ | DNase_I_hypersensitive_site | HSS-1, HSS-2; androgen-stimulated; |
| chr17\|34825294\|34825783\|LTRIS4:ERV1:LTR\|61\|+ | protein_bind: regulator of sex limited protein 1 | ab/bc/cd region; contains multiple RslI binding sites |
| chr17\|34825294\|34825783\|LTRIS4:ERV1:LTR\|61\|+ | enhancer | 750 bp SmaI/XbaI fragment |
| chr17\|34825294\|34825783\|LTRIS4:ERV1:LTR\|61\|+ | promoter | 750 bp SmaI/XbaI fragment |
| chr17\|34825294\|34825783\|LTRIS4:ERV1:LTR\|61\|+ | retrotransposon:LTRIS4 | described in PMID:3167981, PMID:9337392 |
| chr17\|34825294\|34825783\|LTRIS4:ERV1:LTR\|61\|+ | enhancer | del3 fragment |
| chr17\|34825294\|34825783\|LTRIS4:ERV1:LTR\|61\|+ | enhancer | C' delta9 120 bp fragment |
| chr17\|34825294\|34825783\|LTRIS4:ERV1:LTR\|61\|+ | enhancer | C' delta2 160 bp fragment |
| chr17\|34825294\|34825783\|LTRIS4:ERV1:LTR\|61\|+ | enhancer | HRE3 |
| chr17\|34825294\|34825783\|LTRIS4:ERV1:LTR\|61\|+ | protein_bind: androgen receptor | HRE3 |
| chr17\|34825294\|34825783\|LTRIS4:ERV1:LTR\|61\|+ | protein_bind: nuclear receptor subfamily 3, group C, member 1 | HRE3 |
| chr17\|34825294\|34825783\|LTRIS4:ERV1:LTR\|61\|+ | protein_bind: signal transducer and activator of transcription 5 | GAS 1 |
| chr17\|34825294\|34825783\|LTRIS4:ERV1:LTR\|61\|+ | enhancer | HRE2 |
| chr17\|34825294\|34825783\|LTRIS4:ERV1:LTR\|61\|+ | protein_bind: androgen receptor | HRE2 |
| chr17\|34825294\|34825783\|LTRIS4:ERV1:LTR\|61\|+ | protein_bind: runt related transcription factor 2 | Oligo IV |
| chr17\|34825294\|34825783\|LTRIS4:ERV1:LTR\|61\|+ | protein_bind: androgen receptor | HRE1 |
| chr17\|34825294\|34825783\|LTRIS4:ERV1:LTR\|61\|+ | protein_bind: nuclear receptor subfamily 3, group C, member 1 | HRE1 |
| chr17\|34825294\|34825783\|LTRIS4:ERV1:LTR\|61\|+ | protein_bind: POU class 2 homeobox 1 | FPIV |
| chr17\|34825294\|34825783\|LTRIS4:ERV1:LTR\|61\|+ | protein_bind: runt related transcription factor 2 | Oligo III |
| chr17\|34825294\|34825783\|LTRIS4:ERV1:LTR\|61\|+ | protein_bind: nuclear factor of kappa light polypeptide gene enhancer in B cells 1, p105 | FPIII |
| chr17\|34825786\|34826477\|MuRRS4-int:ERV1:LTR\|98\|+ | DNase_I_hypersensitive_site | HSS-1, HSS-2; androgen-stimulated |
| chr17\|34825786\|34826477\|MuRRS4-int:ERV1:LTR\|98\|+ | enhancer | 750 bp SmaI/XbaI fragment |
| chr17\|34825786\|34826477\|MuRRS4-int:ERV1:LTR\|98\|+ | promoter | 750 bp SmaI/XbaI fragment |
| chr17\|34825786\|34826477\|MuRRS4-int:ERV1:LTR\|98\|+ | retrotransposon:MuRRS4-int | imp1 (imposon); described in PMID:3167981, PMID:9337392 |
| chr19\|21164568\|21164695\|B1F:Alu:SINE\|298\|+ | enhancer | VISTA enhancer mm1562 |
| chr19\|21164739\|21164837\|PB1D10:Alu:SINE\|309\|+ | enhancer | VISTA enhancer mm1562 |
| chr19\|21165200\|21165476\|B4:B4:SINE\|337\|+ | enhancer | VISTA enhancer mm1562 |
| chrX\|103477374\|103477481\|B1F1:Alu:SINE\|308\|+ | rep_origin: region spanning amplicons 13 through 15 | region spanning amplicons 13 through 15 |
| chrX\|103477691\|103477768\|ID4:ID:SINE\|250\|+ | rep_origin: region spanning amplicons 13 through 15 | region spanning amplicons 13 through 15 |

**Table S1.** Detailed information of the overlap of Differentially Expressed TEs (“TE”) with RefSeq Functional Elements (“RefSeq Functional Element”).

| **Expression** | **Class** | **Genic proportion** | **Genomic proportion** | **P-value** |
| --- | --- | --- | --- | --- |
| **Up-regulated** | **DNA** | 8% | 4% | 0.0758 |
|  | **LINE** | 18% | 27% | 0.0487* |
|  | **LTR** | 26% | 26% | 1.0000 |
|  | **Other** | 1% | 1% | 1.0000 |
|  | **SINE** | 46% | 42% | 0.3928 |
| **Down-regulated** | **DNA** | 10% | 4% | 0.9340 |
|  | **LINE** | 20% | 27% | 0.8930 |
|  | **LTR** | 70% | 26% | 0.0057* |

**Table S2.** Proportion test results of the selected genic TEs in section 2 of our work versus the genomic TE distribution. The “prop.test” function of the R statistical software was used, and the test p-value results are shown. * denotes changes that are statistically significant at the 0.05 level.

| **Expression** | **Class** | **Intergenic proportion** | **Genomic proportion** | **P-value** |
| --- | --- | --- | --- | --- |
| **Up-regulated** | **LINE** | 16% | 27% | 0.4053 |
|  | **LTR** | 53% | 26% | 0.0200* |
|  | **SINE** | 16% | 42% | 0.0399* |
|  | **DNA** | 15% | 4% | 0.622 |
| **Down-regulated** | **LINE** | 20% | 27% | 1.0000 |
|  | **LTR** | 80% | 26% | 0.0273* |

**Table S3.** Proportion test results of the selected intergenic TEs in section 2 of our work versus the genomic TE distribution. The “prop.test” function of the R statistical software was used, and the test p-value results are shown. * denotes changes that are statistically significant at the 0.05 level.

| **TE** | **Closest Downstream Gene** | **TE distance to gene** | **Closest enhancer to gene** | **Enhancer distance to gene** |
| --- | --- | --- | --- | --- |
| chr7\|98209843\|98210939\|  MTA_Mm-int:ERVL-MaLR:LTR\|35\|+ | Acer3 | 2872 | Alg8/e32113 | 22710 |
| chr7\|98210939\|98211334\|  MTA_Mm:ERVL-MaLR:LTR\|25\|+ | Acer3 | 2477 | Alg8/e32113 | 22710 |
| chr15\|79914180\|79914360\|  URR1A:hAT-Charlie:DNA\|124\|+ | Cbx7 | 1447 | Apobec3/e12617 | 20805 |
| chr15\|79914180\|79914360\|  URR1A:hAT-Charlie:DNA\|124\|+ | Cbx7 | 1447 | Apobec3/e12608 | 20805 |
| chr15\|79914180\|79914360\|  URR1A:hAT-Charlie:DNA\|124\|+ | Cbx7 | 1447 | Apobec3/e12609 | 20805 |
| chr15\|79914180\|79914360\|  URR1A:hAT-Charlie:DNA\|124\|+ | Cbx7 | 1447 | Apobec3/e12610 | 20805 |
| chr15\|79914180\|79914360\|  URR1A:hAT-Charlie:DNA\|124\|+ | Cbx7 | 1447 | Apobec3/e12618 | 20805 |
| chr15\|79914180\|79914360\|  URR1A:hAT-Charlie:DNA\|124\|+ | Cbx7 | 1447 | Apobec3/e12625 | 20805 |
| chr15\|99575851\|99576328\|  RCHARR1:hAT-Charlie:DNA\|248\|- | Faim2 | 47687 | Adcy6/e13236 | 27805 |
| chr6\|134608904\|134609036\|  ID_B1:B4:SINE\|265\|+ | Mansc1 | 171 | Lrp6/e30577 | 42203 |
| chr9\|50508285\|50509045\|  RLTR33:ERVK:LTR\|164\|+ | Pts | 12573 | Plet1/e35851 | 27082 |

**Table S4**. Enhancers within 52 kb of genes associated with intergenic TEs.
