# Supplementary material for: Differential regulation of transposable elements (TEs) during the murine submandibular gland development
Source: Mob DNA. 2021 Oct 22;12:23. doi: 10.1186/s13100-021-00251-1 (PMC8540199; doi:10.1186/s13100-021-00251-1)
Supplement: Supplementary file 6 — Additional file 6: Table S5. Alignment statistics of the RNA-Sequencing samples published by Zhang et al. (2014). Table S6. Alignment statistics of the RNA-Sequencing samples published by Cui et al. (2010). Table S7. Alignment statistics of the RNA-Sequencing samples published by Zhang et al. (2018).. Figure S6. Venn diagram of the TEs identified by SQuIRE in the datasets published by Cui et al. (2010). [file 13100_2021_251_MOESM6_ESM.docx]

**SUPPLEMENTAL INFORMATION 2**

**Differential regulation of TEs during murine Submandibular Gland Development**

Braulio Valdebenito-Maturana, Francisca Torres, Mónica Carrasco^*^, Juan Carlos Tapia^*^

**Information in this document:**

Supplementary methods

Table S5

Table S6

Table S7

Figure S6

**Supplementary methods**

Publicly available data from works comparing the RNA-Sequencing library preparation methods polyA-selection and rRNA depletion were downloaded using *fastq-dump* from the SRA Toolkit (1) (accessions are shown under the “SRA Accession” column of Tables S5, S6 and S7). Then, the FASTQ files were aligned to the *Mus musculus* mm10 or *Homo sapiens* hg38 genomes using STAR v2.7.6 (2). BAM outputs were processed with SAMtools (3) using option -L and the respective BED file of the Transposable Elements annotation to obtain the number of reads aligned to TEs.

SQuIRE was used on the dataset published by (4) to quantify and identify the differences in the TE repertoire between the polyA selection and rRNA depletion library preparation methods.

| **Input RNA** | **SRA Accession** | **Total aligned reads** | **Reads aligned to TEs** | **% of reads in TEs** |
| --- | --- | --- | --- | --- |
| **Mouse ESC, polyA(-)** | SRR1104955 | 7629404 | 563706 | 7.4% |
| **Mouse ESC, polyA(-)** | SRR1104956 | 7954256 | 551729 | 6.9% |
| **Mouse ESC, polyA(-)** | SRR1104957 | 9491195 | 666584 | 7.0% |
| **Mouse ESC, polyA(+)** | SRR1104958 | 11387479 | 2587257 | 22.7% |
| **Mouse ESC, polyA(+)** | SRR1104959 | 12091619 | 2689966 | 22.2% |
| **Mouse ESC, polyA(+)** | SRR1104960 | 11771041 | 2646083 | 22.5% |
| **Mouse hippocampus, polyA(-)** | SRR1104961 | 23776136 | 3135991 | 13.2% |
| **Mouse hippocampus, polyA(+)** | SRR1104962 | 46806730 | 9429458 | 20.1% |

**Table S5.** Alignment statistics of the RNA-Sequencing samples published by (5). ”ESC”: Embryonic Stem Cell lines. “polyA(+)”: polyA selected RNAs for library preparation,

“polyA(-)”: non polyA and rRNA depleted RNAs used for library preparation.

| **Input RNA** | **SRA Accession** | **Total aligned reads** | **Reads aligned to TEs** | **% of reads in TEs** |
| --- | --- | --- | --- | --- |
| **Mouse cerebrum, Ribo-minus** | SRR018013 | 9821530 | 1086257 | 11.1% |
| **Mouse cerebrum, Ribo-minus** | SRR018014 | 9818936 | 1084333 | 11.0% |
| **Mouse cerebrum, polyA mRNA** | SRR018015 | 17965741 | 1400185 | 7.8% |

**Table S6.** Alignment statistics of the RNA-Sequencing samples published by (4).“polyA mRNA”: polyA selected RNAs for library preparation, “Ribo-minus”: rRNA depleted RNAs used for library preparation.

| **Input RNA** | **SRA Accession** | **Total aligned reads** | **Reads aligned to TEs** | **% of reads in TEs** |
| --- | --- | --- | --- | --- |
| **mRNA** | SRR6410603 | 48244986 | 14212727 | 29.5% |
|  | SRR6410604 | 48006711 | 13990315 | 29.1% |
|  | SRR6410605 | 48344945 | 14268154 | 29.5% |
|  | SRR6410606 | 46889771 | 14260819 | 30.4% |
| **Total RNA, Ribo-zero** | SRR6410607 | 46558174 | 8365539 | 18.0% |
|  | SRR6410608 | 48356361 | 8500704 | 17.6% |
|  | SRR6410609 | 48336115 | 8355583 | 17.3% |
|  | SRR6410610 | 48324445 | 8497791 | 17.6% |

**Table S7.** Alignment statistics of the RNA-Sequencing samples published by (6). “mRNA”: mRNA selected for library preparation, “Total RNA, Ribo-zero”: rRNA depleted RNAs used for library preparation.

**
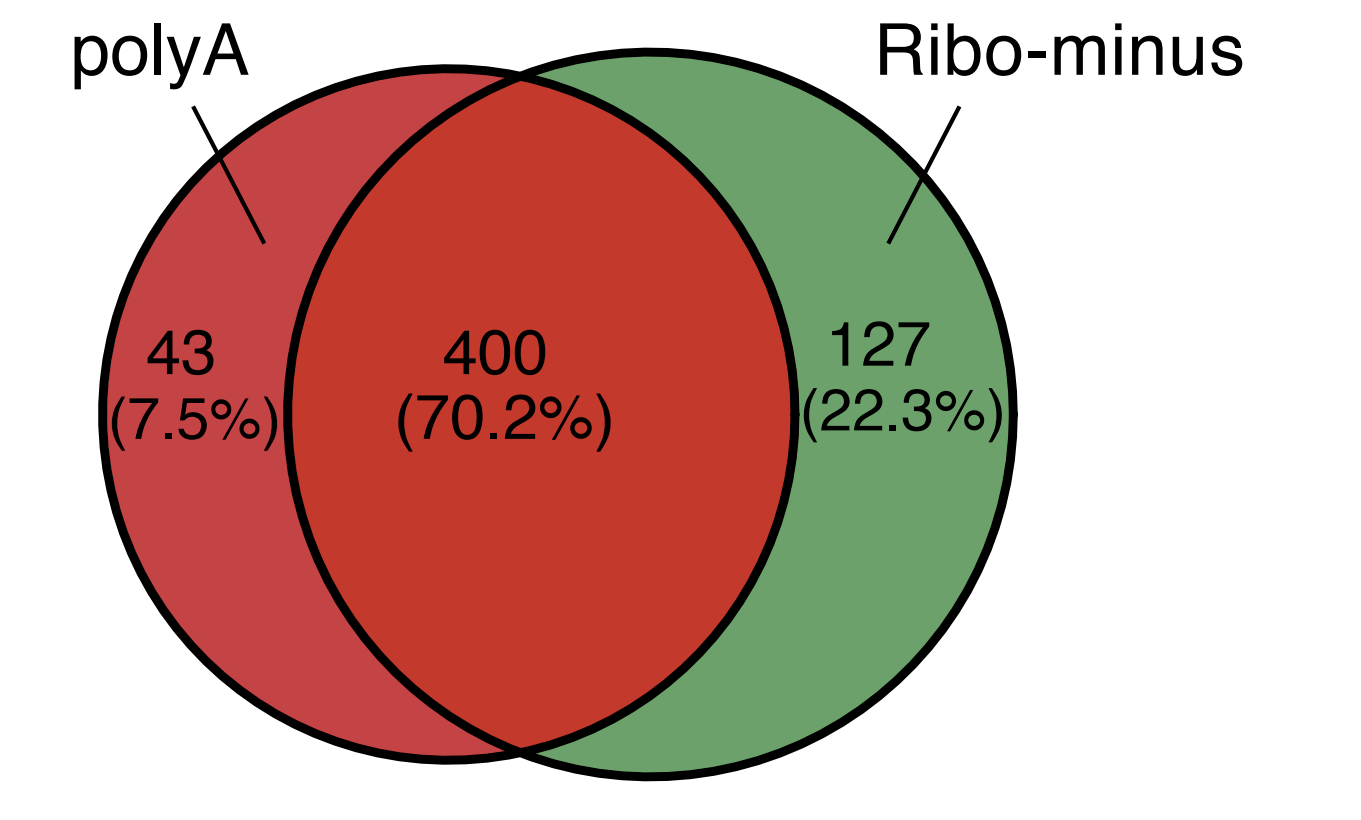
**

**Figure S6.** Venn diagram of the TEs identified by SQuIRE in the datasets published by Cui et al. (2010). “polyA” corresponds to the TEs identified in the polyA-selected library, “Ribo-minus” corresponds to the TEs identified in the rRNA depleted libraries.
